# Ecological Momentary Assessment of Social Contact Satisfaction and Momentary Affect in Chinese Older Adults

**DOI:** 10.1093/geroni/igaa057.2971

**Published:** 2020-12-16

**Authors:** Huiying Liu, Vivian Lou

**Affiliations:** 1 Central South University, Changsha, Hunan, China; 2 The University of Hong Kong, Hong Kong, China

## Abstract

Previous researches have examined the influences of satisfaction with social relations on affective well-being on a long time-frame among older adults. Using the Ecological Momentary Assessment (EMA) method, we examined within-person changes in social contact satisfaction in relation to momentary affect on short time-scales in 78 community-dwelling older Chinese. Each participant provided up to 7 EMA surveys per day during a one-week period and reported his/her satisfaction, positive affect (PA) and negative affect (NA). Multilevel modelling was used for testing the within-person concurrent and time-lagged relations of social interactions and affect. Higher satisfaction was concurrently associated with more PA and less NA. The satisfaction of prior contact was predictive of greater low-arousal PA during the next contact. Prior NA was predictive of lower satisfaction at the next contact. Such dynamic interplays of social and affective experiences should be considered to support the maintenance of affective well-beings in older Chinese.

